# Variation of in-hospital trauma team staffing: new resuscitation, new team

**DOI:** 10.1186/s12873-022-00715-4

**Published:** 2022-09-15

**Authors:** Oscar E. C. van Maarseveen, Roel L. N. Huijsmans, Luke P. H. Leenen, Wietske H. W. Ham

**Affiliations:** Department of Trauma Surgery, University Medical Center Utrecht, Heidelberglaan 100, 3584 CX Utrecht, The Netherlands

**Keywords:** Resuscitation, Trauma team, Composition, Staffing, Variation

## Abstract

**Background:**

Non-technical errors, such as insufficient communication or leadership, are a major cause of medical failures during trauma resuscitation. Research on staffing variation among trauma teams on teamwork is still in their infancy. In this study, the extent of variation in trauma team staffing was assessed. Our hypothesis was that there would be a high variation in trauma team staffing.

**Methods:**

Trauma team composition of consecutive resuscitations of injured patients were evaluated using videos. All trauma team members that where part of a trauma team during a trauma resuscitation were identified and classified during a one-week period. Other outcomes were number of unique team members, number of new team members following the previous resuscitation and new team members following the previous resuscitation in the same shift (Day, Evening, Night).

**Results:**

All thirty-two analyzed resuscitations had a unique trauma team composition and 101 unique members were involved. A mean of 5.71 (SD 2.57) new members in teams of consecutive trauma resuscitations was found, which was two-third of the trauma team. Mean team members present during trauma resuscitation was 8.38 (SD 1.43). Most variation in staffing was among nurses (32 unique members), radiology technicians (22 unique members) and anesthetists (19 unique members). The least variation was among trauma surgeons (3 unique members) and ER physicians (3 unique members).

**Conclusion:**

We found an extremely high variation in trauma team staffing during thirty-two consecutive resuscitations at our level one trauma center which is incorporated in an academic teaching hospital. Further research is required to explore and prevent potential negative effects of staffing variation in trauma teams on teamwork, processes and patient related outcomes.

## Background

The implementation of trauma systems, in conjunction with a systematic approach to trauma resuscitation, has considerably improved the outcome of critically injured patients [1]. One of the pillars of these advances is a coordinated early resuscitation, for which the establishment of in-hospital trauma teams is critical [2–4]. A trauma team’s goal is to diagnose life-threatening injuries and provide immediate resuscitation and stabilization.

Previous studies already found that beside technical failures, non-technical errors, such as insufficient communication or leadership, may hinder these goals and could lead to severe adverse effects, including increased mortality rates [5–9]. Therefore, acquiring non-technical skills is of utmost importance for effective teamwork between physicians, nurses and ancillary personnel in order to accomplish a coordinated resuscitation. Establishing a shared mental model of the patient’s circumstance allows team members from various backgrounds to comprehend both the clinical and logistical implications of individual trauma patients [10, 11]. In short, a shared mental model could be defined as representation of team members’ shared knowledge about the team and its environment, such as the team’s goals, processes, communication, available information and adaptations to situations and members’ roles, behaviors and interactions [12]. The establishment of a shared model is especially important, but also more difficult to achieve, when physicians and nurses from various disciplines converge to resuscitate a critical injured patient under time pressure.

However, little is known about the nature, extent and impact of staffing variation within trauma teams. Intuitively, the more resuscitations performed with the same team members, the more familiar the team members get with each other’s roles, behaviors, and interactions, which facilitates the establishment of a shared mental model. However, based on our own clinical experience, human resources vary considerably and change frequently. Therefore, during resuscitations members of the trauma team sometimes may not even know each other by name. In this study, we aimed to assess the extent of trauma team staffing variability in daily and day-to-day trauma teams. Our hypothesis was that there would be a high variation in trauma team staffing.

## Methods

### Design, sample and outcomes

This study was a retrospective observational study of recorded videos of actual trauma resuscitations. During the a one-week period in May 2018, all successive resuscitations of injured patients were retrospectively examined on trauma team composition utilizing video records. Two experts with significant experience in evaluating video records of trauma resuscitation evaluated all available video recordings. The main outcome of this study was number of unique compositions of trauma teams in terms of personal staffing. Other outcomes were number of unique team members, number of new team members compared to the previous resuscitation and new team members compared to the previous resuscitation in a comparable shift (Day, Evening, Night).

### Setting

#### Trauma center

This study was performed at the University Medical Center Utrecht (UMC Utrecht), an academic teaching hospital and a level one trauma center in the Netherlands. The coordinating emergency department nurse activated the trauma team if one of the preset criteria for admitted trauma patients was met. These criteria are based on the trauma mechanism or specific patient psychological or anatomical conditions as reported by ambulance staff prior to the patients’ admittance. These criteria can be found in Table 1.

**Table 1:** New team members of consecutive resuscitation. Trauma team activation criteria in the University Medical Centre Utrecht. GCS, Glasgow coma score; BSA, body surface area

**Mechanism**	
Motor vehicle accident	•Speed over 80 km/h •Ejection/roll over/trapped •Unrestrained/fatality
Motor bicycle	•Any with speed >30 km/h
Pedestrian/cyclist	•Struck by car or motorcycle/any speed
Fall	•Adult >3 m and/or 5 stairs •Elderly on anti-coagulant therapy •Motor bikes/cycle/water ski
Horse	•Any horse-related injury
Assaults	•Shooting •Stabbing •Focal blunt head trauma with GCS <13
Multiple casualties	•With significant injuries
Other	•Explosion •Hanging •Submersion
**Injuries**	
	•Potential airway obstruction/respiratory distress •Penetrating injury to the head/neck/chest/abdomen/pelvis/back/limbs •Paralysis (spinal cord injury) •Burns >10% BSA
**Signs**	
	•Respiratory rate <10 or >30 •Heart rate <40 or >120 •Arterial pressure <90 systolic at any stage •Capillary return >2 s •GCS <14
**Treatment**	
	•Multi-trauma transferred from other hospital within 24 h of injury •Intubation or assisted ventilation •>2 liter of fluid resuscitation

#### Trauma team composition and activation

The trauma team in the UMC Utrecht has generally the following composition: a trauma surgeon or fellow, a surgical resident, an anesthesiologist’s resident, an emergency physician, a neurologist, two emergency department nurses, and a radiology technician. The article of Kreb et al. [13] provide a more thorough explanation of trauma team composition and task assignment. The trauma surgical resident is the team leader, while the trauma surgeon or resident is directly available, supervise the surgical resident and is ultimately responsible. The emergency physician performs documentation of the resuscitation, contacts the radiologist when a CT scan is required. At the UMC Utrecht, we have a one-tier trauma team activation strategy, which means that the trauma team composition is theoretically the same for each trauma resuscitation. Only the trauma surgeon could decide to consult additional medical doctors or extend the team with an additional nurse. The trauma team is activated in case one of the predefined activation criteria is met. At the time of the study, ten staff trauma surgeons, three trauma surgeons fellows, eleven surgical residents, five ED physicians, roughly 45 nurses could potentially be part of the formation of a trauma team. Yearly there are roughly 1200 trauma admission of which nearly 400 are severely injured.

#### Schedule system

During working days (Monday-Friday) there is a 3-shift system (day, evening, night), for residents and nurses and a 2-shift system for trauma surgeons or fellows. During the weekend there is also a 2-shift system for residents.

### Data collection

First, among all included videos, unique trauma team members were identified and categorized in one of the following groups: trauma surgeon or fellow, surgical resident, emergency physician, anesthesiologist, neurologist, emergency nurse, radiology technician or other. Each video was analyzed to identify attending team members per resuscitation. Resuscitations were categorized in shifts (Day 8.00-16.00; Evening 16.00 -24.00 and Night 24.00-8.00). Data was collected using a score sheet in Microsoft Excel (Microsoft Corp. Released 2010. Microsoft Office Excel 2010, Version 14.0. Redmond, WA: Microsoft Corp.). Finally, resuscitated patient’s baseline characteristics were gathered (age, ISS,).

### Statistical analysis

For this study and SPSS (IBM Corp. Released 2012. IBM SPSS Statistics for Windows, Version 21.0. Armonk, NY: IBM Corp) and Microsoft Excel (Microsoft Corp. Released 2010. Microsoft Office Excel 2010, Version 14.0. Redmond, WA: Microsoft Corp) were used for descriptive analysis. Data was considered nonparametric in case Shapiro-Wilk test’s *p*-values were 0.05 or lower. Baseline characteristics were reported as means and standard deviations (SDs), whilst non-normally distributed data were provided as medians with interquartile ranges (IQRs). Percent deviation (PD) was computed for results reported in percentages.

### Privacy and ethics

This study was approved by the institutional research board. In accordance with the legal department of our hospital, no inform consent of patients nor personnel was needed, as the records of videos are part of our quality assessment program. Video recordings were stored on a secure server inside the hospital building. Video records were automatically removed from the server after fourteen days and data was anonymized within fourteen days.

## Results

### Baseline characteristics

In total, 32 videos of trauma resuscitations were included and analyzed. All provided data were normally distributed (Shapiro-Wilk test *p* > 0.05), except for the ISS. (Shapiro-Wilk test *p* = 0.04). The median ISS was twelve (IQR 5-21) (Table 2).

**Table 2:** Baseline characteristics of resuscitated patients

**Baseline characteristics**	
**Resuscitations, cases**	32
**Age, mean**	50 (SD 18)
**Gender, percentage male**	72
**ISS, median**	12 (IQR 5-21)

### Team staffing variation

All 32 resuscitations had a unique trauma team composition (100%) and a total of 101 unique trauma members were identified (Table 3). Mean number of team members present during trauma resuscitation was 8.38 (SD 1.43). Most variation in staffing was among nurses (32 unique team members), radiology technicians (22 unique team members) and anesthetists (19 unique team members). The least variation was among trauma surgeons (3 unique team members), ER (emergency room) physicians (3 unique team members) and trauma fellows (2 unique team members)

**Table 3:** Team composition of analyzed trauma teams

**Category**	**Unique members (SD)**	**Resuscitations present (%)**
**Total trauma resuscitations**	32	N/A
**Unique trauma teams**	32	N/A
**Team members core team**		
** Trauma surgeon**	3	17 (53.1%)
** Trauma Fellow**	2	11 (29.1%)
** Surgical resident**	7	32 (100%)
** Emergency Physician**	3	13 (40.6%)
** Anesthetist**	19	32 (100%)
** Radiology technician**	22	32 (100%)
** Nurse**	32	32 (100%)
** Neurologist**	11	29 (90.6%)
**Additional team members**		
** Radiologist**	2	2 (6.3%)
**Overall**		
** Total unique trauma team members**	101	
** Average number of members per resuscitation**	8.375 (1.43)	

The mean number of new members in teams of consecutive trauma resuscitations was 5.71 (SD 2.57), which was 67.4% (Percent Deviation (PD) 45.0) of the average total team size (Table 4). Percentage of consecutive resuscitations’ new team members were 64.9% (PD 51.0), 65.5% (PD 38.46), 71.4 % (PD N/A) for day, evening and night shift respectively.

**Table 4:** New team members of consecutive resuscitation

	**Trauma resuscitations (n)**	**Mean new team members compared to previous resuscitation**	
		**N (Standard Deviation)**	**% (Percent Deviation)**
**Total**	32	5.7 (SD 2.6)	67.4 (PD 45.0)
**Day shift (08:00-16:00)**	15	6.0 (SD 3.1)	64.9 (PD 51.0)
**Evening shift (16:00-24:00)**	15	5.3 (SD 2.1)	65.5 (PD 38.5)
**Night shift (00:00-08:00)**	2	5.0 (SD N/A)	71.43(PD N/A)

## Discussion

This is the first study to describe the extent of staffing variety of trauma teams. We found a very high variation in trauma team staffing at our level one trauma center, which is incorporated in an academic teaching hospital. All 32 trauma teams demonstrated an unique composition and 101 unique members were identified of a trauma team. Thereby, we found that on average, two-thirds of the trauma team staffing rotated during the successive resuscitation. Within most academic hospitals, education of (para) medic personnel is common and rotation of residents, fellows and nurses is routine and occurs frequently. As many level one trauma centers are incorporated within academic teaching hospitals variation in trauma teams is likely to be common within trauma resuscitation in the emergency department. In the United States, approximately 75 percent of all level one trauma centers are incorporated in academic teaching hospitals [14].

Some recent studies found positive effects of familiarity of team members on teamwork, processes or patient care, which supports the reasoning that less variation in trauma team staffing may improve trauma care. First of all, Joshi et al. [15] investigated familiarity of team members on teamwork and clinical effectiveness during three simulated trauma scenarios. Teams whose staffing remained constant across the scenarios (stable teams) were compared to team whose staffing fluctuated with each scenario (dynamic teams). 46 trainees (23 General Surgery; 23 Emergency Medicine) were allocated into stable- or dynamic teams. The teamwork in both groups enhanced significantly, but the teamwork was more enhanced in the stable teams (stable: 9%, *p* < 0.04; dynamic = 4.9%, *p* < 0.03). Thereby, significant increased improvements in clinical effectiveness was only seen in the stable team. (stable: 15.2%; *p* = 0.03; dynamic 8.7% *p* = 0.19). A study of Powezka et al. [16] performed a retrospective analysis of 326 vascular procedures. They introduced the Familiarity Score, which yields the total of numbers of times each team member (vascular consultant, vascular registrar, scrub nurse, anesthetic consultant) had worked together, in the previous six months, divided by the number of possible combinations of pairs in the team. They found that the Familiarity Score was significantly associated with the length of the procedure (Bayes Factor= 37). Moreover, Krumann et al, [17] performed a retrospectively analysis on the effect of familiarity among team members on complication rates after elective open abdominal surgery. During a 6- month period a senior and junior surgeon performed all surgical interventions. The first and last month of this period where compared. A significant higher percentage of complications were found during the first period compared to the last period (54.2% vs. 34.5 %; *P* = 0.04), demonstrating familiarity may improve team performance and patient safety. Finally, Obermair and colleagues [18] evaluated impact of team familiarity in elective gynecological surgery on complications among 6,707 medical records. After surgery, the lead surgeon scored familiarity of the team using a five–point Likert scale which was documented at the operation report. In their analyses, after adjustment for ASA score and BMI, the likelihood of an adverse event was doubled in non-familiar teams compared to familiar teams (OR 2.06, 95%CI 1.20 to 3.55 *p* < 0.01). Moreover, in contrast to predictable circumstances during elective surgery or simulated environments, the circumstances during the resuscitations of severely injured patients are more stressful and less predictable, requiring highly adaptive teams. Therefore, extrapolating the findings of the discussed recent studies to actual trauma resuscitations, familiarity may enhance teamwork and team performance even more.

Although this study did not investigate the direct effects of familiarity within trauma teams on patient’s outcome, our findings emphasize the importance of non-technical abilities among team members and clear role assignments of the team members. Nontechnical skills such as communication, leadership, and teamwork are examples of nontechnical skills that are increasingly being recognized as key components of emergency resource management [19]. Thereby, a clear task delineation is required as it is hard to collaborating together without fully understanding each other necessitates. Trauma team simulation training has been proven to increase nontechnical skill development [20]. Furthermore, during simulation training, understanding of role assignment within trauma teams might be a trainable aspect. Therefore, regular trauma team training might reduce the negative effects of unfamiliarity of team members.

### Further research

More research is required to gain insights into the nature and extend of trauma team staffing variations and the impact on patient care and patient outcomes. First of all, to obtain a general overview and to increase generalizability of our study results, our study should be replicated in multiple trauma centers. Second, there is evidence of previous studies that teamwork leads to improved performance [21]. Therefore, we suggest investigating the impact of high variance in team staffing on teamwork. Future research projects, should an may further improve our understanding of the impact of the trauma team variation including clinical outcomes. The overall theory is that considerable variance in trauma team staffing leads to impaired teamwork, which in turn is thought to lead to deteriorated performance. In simplest form, the impact of team variance on teamwork could be assessed during simulation sessions to compare teams with no or little variance to high variance. There are reliable and validated tools available to assess the teamwork, such as the T-NOTECHS tool [22–24]. Third, interventions to effectively reduce team variation could be developed, tested and implemented into practice. We suggest two types of interventions to be investigated. First interventions that reduce team staffing variance and second, interventions that reduce negative effects of team staffing variance. An example how team variance could be reduced is by advanced scheduling systems. Coordination of having the similar staff occupation within teams is extremely challenging, as multiple (para)medical specialties are involved in the trauma team. A possible approach could be scheduling using advanced methods, such as deep learning techniques, as described by Rosemarin [25]. Their supposed deep-learning scheduling system was able to schedule based on hospital’s data and specific goals, which among other goals, could be the reduction of trauma team staffing variance.

### Strengths and limitations

The strength of this study was the use of video recordings of trauma resuscitations to analyze trauma team composition, which provides an unbiased, indisputable and accurate documentation of the trauma resuscitation**.** However, our study also has limitations that should be considered. This was a single-site study in a level one trauma center in an academic institution. The practices and policies at our institution may differ from other academic medical centers and even more from smaller non-academic hospitals. As such, the generalizability of our findings may be limited. Furthermore, we analyzed 32 trauma team activations, within a relative short time. Thereby, most of the trauma resuscitations were during day and evening time, and very few during the night. Therefore, our study populations was too small to perform additional analysis. Therefore, results should be considered as a rough estimation of the extent of staffing variation of trauma teams at our hospital. Nevertheless, we believe that our key finding, that there is a high variance team staffing, will not change with an larger study population. Thereby, theoretically, there shall even be more variations over longer periods, because of rotations of residents, vacations and new personnel during the year.

## Conclusion

We found an extremely high variation in trauma team staffing at our level one trauma center which is incorporated in an academic teaching hospital. Further research is required to explore the nature and impact of high variation in trauma team staffing on teamwork, processes and patient related outcomes.

## Data Availability

The datasets generated and/or analysed during the current study are not publicly available due to privacy regulations, but are available from the corresponding author on reasonable request.

## Abbreviations

ASA Score: American Society of Anesthesiologists Score
BMI: Body Mass Index
ER physicians: Emergency Room physicians
IQR: Inter Quartile Range
PD: Percent Deviation
SD: Standard deviation
T-NOTECHS: Trauma Non-Technical Skills
UMC Utrecht: University Medical Center Utrecht

## References

[1] Celso B, Tepas J, Langland-Orban B, Pracht E, Papa L, Lottenberg L (2006). A systematic review and meta-analysis comparing outcome of severely injured patients treated in trauma centers following the establishment of trauma systems. J Trauma.

[2] Driscoll PA, Vincent CA (1992). Organizing an efficient trauma team. Injury.

[3] Adedeji OA, Driscoll PA. The trauma team--a system of initial trauma care. Postgraduate medical journal. 1996;72(852):587–93. 10.1136/pgmj.72.852.587 PMC2398610 8977939

[4] Gondek S, Schroeder ME, Sarani B (2017). Assessment and resuscitation in trauma management. Surg Clin North Am.

[5] Uramatsu M, Fujisawa Y, Mizuno S, Souma T, Komatsubara A, Miki T (2017). Do failures in non-technical skills contribute to fatal medical accidents in Japan? A review of the 2010–2013 national accident reports. BMJ Open..

[6] Künzle B, Kolbe M, Grote G. Ensuring patient safety through effective leadership behaviour: a literature review. Safety Science. 2010;48(1):1–7.

[7] Manser T (2009). Teamwork and patient safety in dynamic domains of healthcare: a review of the literature. Acta Anaesthesiol Scand.

[8] Chreiman KM, Dumas RP, Seamon MJ, Kim PK, Reilly PM, Kaplan LJ, et al. The intraosseous have it : a prospective observational study of vascular access success rates in patients in extremis using video review. 2018;84(4):558–63. 10.1097/TA.0000000000001795 PMC5860964 29300281

[9] Nikouline A, Quirion A, Jung JJ, Nolan B (2021). Errors in adult trauma resuscitation: a systematic review. Can J Emerg Med.

[10] Westli HK, Johnsen BH, Eid J, Rasten I, Brattebø G. Teamwork skills, shared mental models, and performance in simulated trauma teams: an independent group design. Scand J Trauma Resusc Emerg Med. 2010. 10.1186/1757-7241-18-47 PMC2939527 20807420

[11] Johnsen BH, Westli HK, Espevik R, Wisborg T, Brattebø G (2017). High-performing trauma teams: frequency of behavioral markers of a shared mental model displayed by team leaders and quality of medical performance. Scand J Trauma Resusc Emerg Med.

[12] Lim B, Klein KJ (2006). Team mental models and team performance: a field study of the effects of team mental model similarity and accuracy. J Organ Behav.

[13] Tiel Groenestege-Kreb D, van Maarseveen O, Leenen L (2014). Trauma team. Br J Anaesth.

[14] MacKenzie EJ, Hoyt DB, Sacra JC, Jurkovich GJ, Carlini AR, Teitelbaum SD (2003). National Inventory of Hospital Trauma Centers. J Am Med Assoc.

[15] Joshi K, Hernandez J, Martinez J, AbdelFattah K, Gardner AK (2018). Should they stay or should they go now? Exploring the impact of team familiarity on interprofessional team training outcomes. Am J Surg.

[16] Powezka K, Normahani P, Standfield NJ, Jaffer U (2020). A novel team familiarity score for operating teams is a predictor of length of a procedure: a retrospective bayesian analysis. J Vasc Surg.

[17] Kurmann A, Keller S, Tschan-Semmer F, Seelandt J, Semmer NK, Candinas D (2014). Impact of team familiarity in the operating room on surgical complications. World J Surg.

[18] Obermair A, Simunovic M, Janda M (2020). The impact of team familiarity on surgical outcomes in gynaecological surgery. J Obstet Gynaecol.

[19] Briggs A, Raja AS, Joyce MF, Yule SJ, Jiang W, Lipsitz SR (2014). The impact of trauma team training on team leaders’ non-technical skills during simulated trauma scenarios. J Surg Res.

[20] Gjeraa K, Møller TP, Østergaard D (2014). Efficacy of simulation-based trauma team training of non-technical skills. A systematic review. Acta Anaesthesiol Scand.

[21] Schmutz JB, Meier LL, Manser T (2019). How effective is teamwork really? The relationship between teamwork and performance in healthcare teams: a systematic review and meta-analysis. BMJ Open.

[22] van Maarseveen OEC, Ham WHW, Huijsmans RLN, et al. Reliability of the assessment of non-technical skills by using video-recorded trauma resuscitations. Eur J Trauma Emerg Surg. 2022;48:441–47. 10.1007/s00068-020-01401-5 PMC8825620 32617607

[23] Steinemann S, Berg B, DiTullio A, Skinner A, Terada K, Anzelon K (2012). Assessing teamwork in the trauma bay: introduction of a modified “NOTECHS” scale for trauma. Am J Surg.

[24] Stevenson C, Bhangu A, Jung JJ, MacDonald A, Nolan B. The development and measurement properties of the trauma NOn-TECHnical skills (T-NOTECHS) scale: a scoping review. Am J Surg. 2022. 10.1016/j.amjsurg.2022.05.027 35659768

[25] Rosemarin H, Rosenfeld A, Kraus S (2019). Emergency department online patient-caregiver scheduling. Proceedings of the AAAI Conference on Artificial Intelligence.

